# Analyzing Psychotherapeutic Failures: A Research on the Variables Involved in the Treatment With an Individual Setting of 29 Cases

**DOI:** 10.3389/fpsyg.2019.01250

**Published:** 2019-06-04

**Authors:** Simone Maggio, Sara Molgora, Osmano Oasi

**Affiliations:** Department of Psychology, Catholic University of the Sacred Heart, Milan, Italy

**Keywords:** psychotherapeutic treatments, psychotherapeutic alliance, psychotherapeutic failures, countertransference, dropout

## Abstract

The effectiveness of psychotherapeutic treatments has been widely demonstrated and confirmed by many studies in recent decades. The research focused on the factors of change influencing the positive outcomes of a psychotherapy, putting those that are crucial in cases of failure into the background. The dimensions of this phenomenon are relevant as well as the side effects of the psychotherapeutic interventions that reach the same percentages of the pharmacotherapeutic treatments. The study of the variables involved in failure cases therefore seems important to prevent or moderate the negative effects of treatments with a negative outcome. Impasse and deadlock situations, which may result in an early interruption of psychotherapy, are often complex and involve situational, relational, and personal factors at different levels and with different weight. A research was conducted, with a mixed approach, aimed at exploring the situational factors involved in dropout cases. In addition, the evaluation of the psychotherapist’s emotional responses related to patients who terminated psychotherapy prematurely was investigated. The study was attended by a sample of 29 psychologists, experienced psychotherapists from different frameworks. Recent or salient cases of a hesitated psychotherapy with an early interruption were examined. For the first objective, a structured interview (Impasse Interview) was used, while the second one was reached by the administration of the TRQ (Therapist Response Questionnaire). The transcripts of the interviews were analyzed through a textual analysis software and five salient thematic clusters were identified. These were then assimilated to different areas of meaning: severity of the diagnosis, procedural aspects and lack of understanding of the stall in progress. Two other important themes emerged: the critical aspects concerning relational dynamics and a focus on maternal theme. Overall these five thematic areas seem to play an important and specific role compared to dropout cases. Finally, statistical analysis on emotional responses have highlighted some values above the average in these four countertransference factors: Helpless/Inadequate, Parental/Protective, Positive/Satisfied, and Overwhelmed/Disorganized. It is hypothesized that particular emotional responses of the psychotherapist may be prognostic with respect to the outcome of psychotherapy.

## Introduction

Linden (2013) states that psychotherapy is considered a treatment modality that has positive effects and that both risks and side effects are limited (Nutt and Sharpe, 2008). However, as shown by various studies, the undesirable effects of psychotherapies treatments range between 3 and 15% of cases; percentages similar to those found for the side effects of pharmacotherapeutic treatments (Mays and Franks, 1985; Mohr, 1995; Roback, 2000; Moos, 2005; Boisvert and Faust, 2006; Jarrett, 2007; Berk and Parker, 2009). The premature conclusion of treatment is generally considered a critical element for the provision of services dedicated to mental health (Wierzbicki and Pekarik, 1993); indeed, in cases of premature abandonment there is a reduction in the effectiveness of the treatment and the cost-benefit ratio is also reduced (Pekarik, 1985; Garfield, 1986). One of hand, while the efficacy of psychological and psychotherapeutic treatment has been solidly confirmed by numerous studies (Roth and Fonagy, 1996, 2004; Nathan and Gorman, 1998, 2007; Kazdin and Weiss, 2003), as Barlow (2010) emphasize, on the other hand, the analysis of the negative outcomes of psychotherapy has been dealt with more recently. Nevertheless, this is still not enough (Linden, 2013), especially considering the need to take into consideration many variables. They can refer not only to the characteristics of the patient or the therapist (for example, age or diagnosis of the patient), but also to elements external to them (for example, public or private setting) (Edlund et al., 2002; Schottenbauer et al., 2008; Olfson et al., 2009; de Haan et al., 2013).

The meta-analysis by Kächele and Schachter (2014) compared to the size of the dropout phenomenon, i.e., the premature termination of therapy, shows percentages that oscillate between 32% of the study of Sledge et al. (1990) of psychotherapy with a limited duration and 67% for short psychotherapies (Sledge et al., 1990). The average percentage of dropouts of all the studies presented by Kächele and Schachter (2014) is in the order of 48%. In another meta-analysis on 11 researches on individual treatments of adults terminated with a dropout, Sharf et al. (2010) highlight (1) how there is a moderately strong relationship between therapeutic alliance and abandonment of therapy and (2) such as patients with a weaker therapeutic alliance have a higher chance of terminating therapy with a dropout. Moreover, the emotional response of the therapist during the treatment plays an important role in the dropout: the Frayn’s (1992) study shows a significant correlation between the patient’s abandonment of therapy and the attitudes of fear, hostility, and worry of the therapist.

On a more general level, an analysis of the literature gives an idea of the factors associated with the impasse (Weiner, 1974; Watkins, 1983; Taylor, 1984; Grunebaum, 1986; Atwood et al., 1989; Mordecai, 1991; Elkind, 1992; Nathanson, 1992; Pulver, 1992; Strupp, 1993; Omer, 1994; Newirth, 1995); Hill et al. (1996) aggregated them highlighting how, according to clinicians, impasse situations can be linked to:

- Pathology of the client, which prevents the latter from being able to benefit from the treatment.
- Contrasts between patient and therapist caused by the respective periods of life, different personalities, theoretical orientation or ultimately personal issues and preferences.
- Problematic aspects of the therapeutic relationship, as a weak to the therapeutic alliance, a rigid or unrecorded relationship or an infringement in the attachment bond.
- Failure to agree on the goals of the therapy or a failure in the communication of the same.
- Patient transference or inappropriate gratification of the patient.
- Countertransference of the therapist or personal issues that interfere with their ability to adequately deliver therapy.
- Errors of the therapist such as: a wrong diagnosis, acting-out, inappropriate interventions, collusion, pejorative communication or even the non-recognition of the goals achieved or reachable by the patient.
- Feelings of the patient’s shame in addressing some issues related to cultural reasons.
- Irreconcilable conflicts and power struggles.
- Real issues related to the situation or external, such as the death of a relative.

In a few years’ work, Kächele and Schachter (2014) report a list of factors generally associated with therapeutic failures, taken from a work by Stein (1972). Here they are:

- Incorrect diagnosis with the consequent administration of an unsuitable treatment.
- Inappropriate external conditions; such as those in which it is noted that external conditions are so unfavorable that it seems preferable to maintain a morbid state rather than to heal. Still those in which the behavior of the family supports every neurotic or psychotic manifestation of the patient. And finally, a series of real factors such as education, social class, economic status, and the effects of a trauma such as illness or mourning.
- Constitutional factors of the patient.
- Unwanted changes of the patient’s ego with relapses in terms of personality disorder.
- Aspects related to transference and countertransference.

According to Kächele and Schachter (2014) the most neglected factor in almost all psychotherapies is countertransference, confirming the position of Frayn (1992) and pointing out that it is in fact the only factor significantly attributable to the therapist present in the list just mentioned. A study of single cases (Bergin and Strupp, 1972) and an idiographic approach, compared to the nomothetic one, seems more profitable than the case study methodology of bankruptcy cases. According to Barlow (2010) this method allows a series of advantages from the point of view of results, namely: (1) prevents important data from being diluted in group averages and (2) allows to identify cause/effect relationships for negative outcomes. As reported by Berk and Parker (2009), a longitudinal and retrospective approach would also give greater possibilities to discern specific factors from non-specific factors linked to negative outcomes.

As mentioned above, the therapist’s reactions to the patient, whether conscious, unconscious, emotional and cognitive, internal or external, can be useful in diagnosis but can also negatively or positively influence the course of treatment (Winnicott, 1949; Heimann, 1950). According to Betan et al. (2005) the therapist’s responses to the patient could provide an *in vivo* understanding of the patient’s relational patterns. The patient could indeed inspire in the therapist those feelings that he is not able to recognize (Klein, 1946), but he could also urge the clinician to put into practice the agitation consistent with his expectations regarding the relationship (Ogden, 1982; Gabbard, 2001). In this sense, the concept of role-responsiveness, proposed by Sandler (1976), makes reference to the fact that the therapist acts coherently with the patient’s relational paradigms, re-proposed by the latter in the psychotherapeutic relationship. Therefore, another aspect related to the phenomenon of the transference, is the one which sees the patient manipulating or provoking situations that are a concealed re-issue of past relationships and experiences with others (Sandler et al., 1973).

In order to operationalize the countertransference construct, Betan et al. (2005) have put in place the TRQ (Therapist Response Questionnaire or Countertransference Questionnaire). It is a tool that aims at evaluating the cognitive, affective, and behavioral response of a clinician in the interaction with a specific patient. This tool has been realized starting from a revision of the clinical, theoretical, and empirical literature on the concept of countertransference and the items it is composed of have been formulated with a common language and therefore they are used by clinicians who refer to clinical and theoretical different approaches. The TRQ identifies eight possible countertransference dimensions that can be stimulated by the patient during psychotherapeutic treatment. They represented the different reactions that therapists can have toward patients and that probably reflect a mixture of the therapist’s own dynamics, responses evoked by the patient and by therapist-patient interactions. Furthermore, the different countertransference factors show a significant association with the DSM-IV TR cluster A, B, and C, which classify personality disorders; in particular, cluster A is correlated with the Criticized/Devalued countertransference, but it is not correlated with the Disengaged countertransference; cluster B is correlated with the countertransference Overwhelmed/Disorganized, Helpless/Inadequate, Disengaged, and Sexualized and is negatively correlated with the positive countertransference; cluster C is correlated with the Parental/Protective countertransference. What the authors point out is that the countertransference framework is very complex and more nuanced than a generic distinction between positive and negative countertransference. The significant correlations between the eight transference dimensions and the symptoms that characterize personality disorders show that the therapist’s emotional responses are expressed in coherent and predictable patterns (Betan et al., 2005).

Therefore, not only do patients evoke specific responses in the therapist, related to his personal history and to the interaction in therapy, but also they elicit an average predictable response and probably similar to that of others important people in their life. The correlation between the activation of specific countertransference dimensions and the characteristics of the different personality disorders makes the countertransference very useful for a diagnostic understanding of the patient’s dynamics and of the repetition of certain relational patterns (Betan et al., 2005).

## Materials and Methods

### Aims of the Study

(1) Analyze the transcripts of the interviews, questioning the possible variables identified by the psychotherapist as causes of the dropout.
(2) Explore the linear associations between specific countertransference responses and dropouts.

### Research Hypothesis

(1) It is expected that dropout cases are positively correlated with specific countertransference responses.
(2) It is expected that there are significant differences among therapist’s countertransference responses, according to some structural variables related to the therapist (gender and orientation) and to the patient (gender and diagnosis).

### Stages of Research

(1) 2018, February – March. Participants recruitment.
(2) 2018, April – May. Conducting interviews and data collection.

### Sample Recruitment

The participants in this research were recruited through four modalities (1) informal network of contacts (2) the Google search engine (3) two professional sites gathering profiles with curricula and services offered by psychologists and psychotherapists: https://www.psicologi-italia.it and https://www.guidapsicologi.it/finally (4) Facebook group composed by psychologists and psychotherapists: https://www.facebook.com/groups/854281581289546/.

The participants were contacted both by telephone and by e-mail address. Later, an e-mail was sent to the therapists contacted by telephone with the request for participation in the research with an interview *de visu*. For the others, the e-mail was sent directly with the request for participation. To avoid possible bias, detailed information on the research design was provided at the end of the meeting. Before the interview, each participant was asked to sign the informed consent form and was asked to record the audio of the interview; all the participants agreed to both requests.

At the end of the interview, each participant was asked to fill in the TRQ with reference to the case illustrated during the interview; at the end, information and details regarding the research design were provided in a brief debriefing; only in one case the TRQ was completed after the meeting and withdrawn after 1 week from the interview. In another case only the interview was carried out, but it was not possible to administer the TRQ. In one case, the question n° 22 of the fourth section of the interview and, in another case, the question n° 20 of the fourth section of the interview could not be formulated.

### Sample of Therapists

The sample consists of a total of 29 psychologists – psychotherapists, among with there are 22 female and 7 male; 28 of these are Italian and 1 is Spanish. The average age is 44.7 years (SD = 9.2) with a range of 34–76 years. All recruited participants are registered in the professional register of psychologists and have been annotated in the Register of Specialization in Psychotherapy for at least 5 years. The general sample is composed of three subgroups that differ according to the psychotherapeutic orientation: Group 1 is formed by psychodynamic psychotherapists (*N* = 10), Group 2 by cognitive-behavioral guidance psychotherapists (*N* = 9), and Group 3 by psychotherapists to orientations not related to the first two (*N* = 10).

### Dropout Cases

The sample of patients, examined in this survey and whose individual therapy ended with a dropout, is 29 subjects; of these 16 of females and 13 of the male gender. Compared to the diagnosis, referring to the DSM-IV TR, 17 patients had a symptomatology attributable to Axis I, 11 patients a disorder attributable to Axis II and 1 patient with no diagnosis. For 27 patients the setting was the private study, whereas for 1 patient the therapy was provided through an advisory service and for another one the therapy was provided via teleconference through the Skype software.

The average number of weekly sessions provided is 1.07; the number of sessions delivered ranges from 2 to 640 sessions (median = 26, *M* = 55.79, SD = 119.18) for a period ranging from 1 to 108 months (median = 6, *M* = 15.08, SD = 25.60). Sessions characterized by an impasse situation range from 0 to 80 (median = 3, *M* = 6.67, SD = 14.41).

### Instruments

#### Impasse Interview

Hill et al. (1996) have put The Questionnaire On Impasse into Individual Therapy, a self-report tool to compile paper and pen, developed from a review of the literature on stalemates in psychotherapy and from the Rhodes et al. (1994) questionnaire. The questionnaire retrospectively investigates a salient or recent case that occurred to the terminated therapist. The questionnaire consists of four sections: (1) General information about the therapist, regarding his training and his psychotherapeutic orientation (2) general information on the situations of impasse experienced by the therapist (3) general information about the patient involved in recent or salient impasse situation (4) in-depth analysis of the impasse with the chosen patient.

The definition of impasse proposed to the therapists was that of a situation of difficulty or stalemate that leads the therapy to become so difficult and complicated as to make it impossible to progress and to cause an interruption. Furthermore, the impasse situation was accompanied by feelings of anger, disappointment, or sense of failure on the part of the therapist or patient.

For the present study, the questionnaire by Hill et al. (1996) was translated into Italian, revised by a doctor in English mother tongue psychology, and culturally adapted to the Italian context. The original questionnaire was then reshaped into a structured interview with the same four thematic sections; no questions have been added or deleted from the Hill et al. (1996).

#### Therapist Response Questionnaire

The Italian version of the TRQ (Zittel Conklin and Westen, 2003; Betan et al., 2005) has been translated and validated by Tanzilli et al. (2016). Like the original version, it is composed of 79 items that investigate a wide range of thoughts, feelings and behaviors of the therapist toward the patient. Compared to the version of Betan et al. (2005) in the Italian version there was the introduction of a ninth factor, obtained through the split of the Criticized/Mistreated factor in: “Criticized/Devalued” and “Hostile/Angry.” The Hostile/Angry factor refers to items that indicate anger, hostility and irritation toward the patient. In the Italian version an analysis was also performed to verify the correlation between the nine factors and the specific personality disorder with the SWAP – 200 scales (Westen and Shedler, 1999a,b; Shedler and Westen, 2004, 2007) in the version Italian by Shedler et al. (2014); in the version of Betan et al. (2005) was carried out only at the level of Clusters A, B, and C. The criterion validity test showed a strong significance between the therapist’s response and the patient’s personality disorder.

### Data Analysis

The questionnaire was analyzed performing statistical analyzes with SPPS 22.0 for Windows (IBM, Armonk, NY, United States). Descriptive, correlational, and *post hoc* analysis were performed.

The interview was audio-recorded and then transcribed electronically using an online transcription software and its application ^1^. The transcriptions were subsequently supervised in analog mode. The *verbatim* of the interviews was analyzed with T-LAB (version 7.3.0; Lancia, 2004). It is a Computer Assisted Data Qualitative Analysis Software (CAQDAS), based on a mixed-method (i.e., quantitative and qualitative) and consisting of a set of linguistic, statistical and graphical tools, that allow, through several algorithms, to carry out different operations both an exploratory as well as an interpretative level to deepen the texts. In particular, it allows to evaluate the relations among words (i.e., lexical units) within a entire text (i.e., the *corpus*), or within specific sections of the text (i.e., the elementary context – that is, the segmentation of the corpus automatically done by the software, or the context units – that is, the segmentation of the corpus done by the researcher on the basis of some independent variables). Unlike theory-driven software, that store information produced by the researcher and return it in an orderly manner, T-LAB is a word-driven software able to create new data (e.g., occurrence and co-occurrence matrices). They have to be interpreted. In this perspective, the software T-LAB, combining linguistics and statistics, offers advantages in term of rigor and reliability of the analyses (Lancia, 2004).

In this study, the corpus was composed by the 29 interviews. Before starting the analyses, the corpus needs to be cleaned, following the rules of cleaning and adaptation of the text, as foreseen by the developers of the software. For the aims of this study, sections 3 and 4 of each interview were used, i.e., those that concerned the chosen case of dropouts in its general aspects and then specific with respect to what happened. Specifically, three different analyses were performed: the thematic analysis of elementary contexts, the correspondence analysis and the specificity analysis. The thematic analysis of the elementary contexts allows to build a representation of the corpus content through the identification of significant thematic clusters (from a minimum of 3 to a maximum of 50): each cluster consists of a set of elementary contexts (i.e., sentence or paragraph) characterized by the same keywords patterns and is described through the lexical units and variables that most characterize the elementary contexts of which it is composed. The result of these analyzes allows a mapping of general or specific themes characterized by the co-occurrence of semantic traits.

The correspondence analysis allows to detect the similarities and the differences among the context units; in particular with respect to the words for categories of a variable with occurrence values. Similar to factor analysis, this analysis extracts a set of new variables (i.e., factors), each of them setting up a spatial dimension on the negative and positive endpoints: the elements (levels of variables and words) that are placed on opposite ends of the factor are most different from each other. Finally, the specificity analysis allows to identify which lexical units are typical (i.e., statistically over-used) or exclusive in a portion of the corpus identified by a categorical variable. Both the correspondence analysis and the specificity analysis are comparative analyses that allow to make a comparison among different segments of the corpus: the first one is possible only with variables that have at least three levels, while the second one can be performed also with two levels variables. In our study, these following independent variables were examined:

(1) Orientation of the therapist: Psychodynamic, Cognitive-behavioral and Other orientations (three levels).
(2) Diagnosis: Axis I, Axis II, and No Diagnosis (three levels).
(3) Post dropout supervision: Yes, No (two levels).
(4) Method of interruption of treatment: *de visu* (communicated during a session), mediated (through a telephone communication or with a mobile phone message), therapist (when the therapist communicates the impossibility of continuing treatment), and none failure to communicate of the end of therapy by the patient (four levels).
(5) Triangulation (Hill et al., 1996): Yes, No (two levels).

## Results

Twenty-eight therapists filled in the TRQ. It is made up of 21 females and 7 males. The average age of the group is 44.9 years (SD = 9.3), with a range from 34 to 76 years. With respect to orientation, 10 therapists had a psychodynamic approach, 9 a cognitive-behavioral approach and 9 referred to other approaches. The group of dropout cases examined is therefore 28 patients, of which 13 (46.4%) of the male gender and 15 (53.6%) of the male gender. Compared to the diagnosis, 16 (57.1%) of these patients reported symptoms referring to Axis I, 11 (39.3%) to Axis II and 1 (3.6%) no diagnosis.

### Emotional Responses of Therapists in Dropout Cases

The mean values of the sample compared to the nine countertransference dimensions are reported in Table 1. In particular, we have indicated the overall values; the values by the therapist’s gender (second and third columns); the values by the patient’s gender (fourth and fifth columns); the values by the patient’s diagnosis (sixth and seventh columns).

**Table 1:** Mean values of the nine countertransference of the TRQ.

Types of controtransfert	Overall mean (SD)	T. men mean (SD)	T. women mean (SD)	P. men mean (SD)	P. woman mean (SD)	Axis 1 mean (SD)	Axis 2 mean (SD)
Helpless/Inadequate	2.81 (0.93)	1.86 (0.60)	3.13 (0.80)	2.61 (1.00)	2.98 (0.86)	2.50 (0.76)	3.27 (1.03)
Parental/Protective	2.78 (0.91)	2.66 (0.83)	2.83 (0.95)	2.57 (1.03)	2.98 (0.78)	2.91 (0.93)	2.51 (0.85)
Positive/Satisfying	2.59 (0.62)	2.71 (0.32)	2.55 (0.69)	2.56 (0.46)	2.62 (0.73)	2.72 (0.42)	2.37 (0.81)
Overwhelmed/Disorganized	2.35 (0.90)	1.80 (0.57)	2.55 (0.92)	2.04 (0.65)	2.62 (1.02)	1.95 (0.57)	2.90 (1.01)
Criticized/Devalued	2.14 (0.77)	1.71 (0.53)	2.29 (0.80)	1.84 (0.67)	2.41 (0.78)	1.82 (0.55)	2.65 (0.84)
Hostile/Angry	2.08 (0.63)	1.88 (0.38)	2.15 (0.70)	2.00 (0.66)	2.14 (0.63)	1.81 (0.54)	2.43 (0.62)
Disengaged	1.88 (0.60)	1.66 (0.50)	1.94 (0.63)	1.89 (0.66)	1.85 (0.57)	1.81 (0.57)	1.87 (0.63)
Special/Overinvolved	1.68 (0.56)	1.74 (0.80)	1.66 (0.48)	1.43 (0.44)	1.89 (0.58)	1.80 (0.65)	1.53 (0.38)
Sexualized	1.33 (0.62)	1.39 (0.57)	1.31 (0.65)	1.58 (0.82)	1.12 (0.25)	1.39 (0.66)	1.11 (0.38)

The therapist’s gender differences, as well as the patient’s gender and diagnosis differences by the therapist’s emotional responses and specific countertransference factor were explored using several *t*-test for independent samples, applying the Bonferroni correction (*p* < 0.005). With respect to the therapist’s gender and to the patient’s diagnosis no significant differences emerged: the mean values do not differ significantly neither between male and female therapists nor between patients with Axis I or Axis II diagnosis. However, the imbalance between men and women requires to consider the results related to the gender of the therapist still provisional. On the contrary, with respect to the patient’s gender, a significant difference emerged for the Sexualized countertransference with the female group showing a lower average than the group of male patients [*t*_(26)_ = 2.06, *p* = 0.000]. For all other factors the mean values do not differ significantly.

Finally, the therapist’s orientation differences by the emotional responses and specific countertransference factor were explored using an one-way ANOVA, applying the Bonferroni correction (*p* < 0.005). Findings show that the psychotherapeutic orientation has not a significant influence on any of the countertransference factors, that is there are not specific countertransference styles depending on the therapist’s orientation.

### Results of Textual Analysis of Transcripts With T-LAB

Compared to the variables examined for this analysis, the results of the group of therapists show among them 15 (5 males, 10 females) who did not carry out post-dropout supervision while 14 (2 males, 12 females) instead performed it. Regarding the Triangulation variable in 23 cases the presence was not detected, in six cases it was present. Compared to the Mode of Communication of the interruption of therapy, in 10 cases it was communicated by the patient *de visu*, in 8 cases, instead, it occurred by telephone with a message or a call. Furthermore, in three cases it was the therapist to report to the patient the interruption of the therapy and in eight there was no communication of the interruption by the patient.

### Thematic Analysis of Elementary Contexts

**FIGURE 1:**
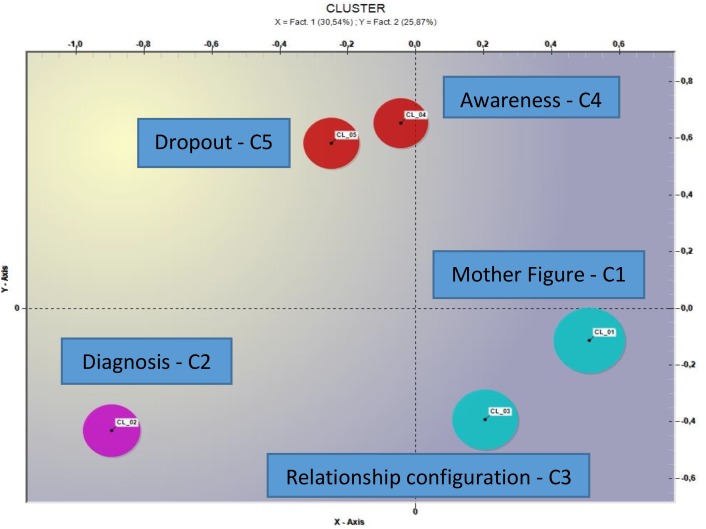
Representation of the spatial distribution of the five thematic clusters.

The thematic analysis of elementary contexts with the use of the measure of the cosine through the bisecting method K-means (Savaresi and Booley, 2001), implemented on the whole corpus of the 29 interviews, produced a five thematic cluster solution (the distribution in the factorial space is shown in Figure 1).

- Cluster 1, labeled *Mother Figure*, aggregates 446 elemental contexts of the 1682 classified, which correspond to 25.52% of the variance (see Table 2).

**Table 2:** Thematic analysis of elementary contexts.

Cluster 1		Cluster 2		Cluster 3		Cluster 4		Cluster 5	
Lemma	χ^2^	Lemma	χ^2^	Lemma	χ^2^	Lemma	χ^2^	Lemma	χ^2^
She	562.22	Years	252.47	To ask	199.30	To understand	142.33	Impasse	106.62
Receive	36.85	Diagnosis	84.01	To take	126.03	Probably	95.03	Before	64.64
Alone	36.85	Disorder	71.54	Appearance	68.40	To tie	88.13	To end	60.16
Search for	34.46	Rating	68.88	Work	52.66	Bound	72.67	Last	58.49
To write	31.50	Anxiety	59.46	Time	46.71	Path	55.17	Of course	51.65
To imagine	29.67	Way	58.66	To happen	43.04	Happen	53.74	You	43.24
Mom	26.17	I remember	58.06	Much more	39.90	_interv_27	42.78	Sitting	37.77
To resume	25.82	Recent	44.64	Boy	37.19	Respect	40.29	Thing	35.09
Series	25.21	To dream	41.03	Therapeutic_alliance	36.07	Sense	37.45	May	31.20
Reality	24.58	Axis	35.75	Ok	33.57	_interv_6	35.57	Coping	30.75
Mother	23.00	Origins	35.75	Decision	30.34	Persistent	32.93	Best wishes	30.12
_interv_19	22.80	Personality_disorder	34.82	Report	26.02	Usual	32.69	Guide	30.12
Answer	21.68	Novo	34.82	Good	24.10	Aloof	27.44	To shift	29.67
Return	21.68	Anxious	34.57	Know	23.83	Bag	26.54	Go out	28.88
_supervis_yes	21.55	Meetings	32.07	Schedule	22.91	Past	26.32	To consider	26.94
- The presence of words like *she*, *mom*, *mother* evokes the reference to a mother figure; moreover, the terms *alone*, *search for*, *to imagine* connote this female figure with respect to experiences of absence or difficulty.
- Cluster 2, named *Diagnosis*, aggregates 303 elementary contexts of the 1682 classified, which correspond to 18.01% of the variance (see Table 2).
- In this cluster the recurrence of the terms *diagnosis*, *disorder*, *evaluation*, *axis*, and *origins* refer to a purely diagnostic theme linked therefore to the patient’s pathology and to its evaluation. The recurrence with the word *Axis II*, *Personality Disorder* refers to a specific type of psychological distress.
- Cluster 3, named *Relationship Configuration*, aggregates 395 elemental contexts of the 1682 classified, which correspond to 23.48% of the variance (see Table 2).
- The presence of terms such as *to ask*, *to take*, *know*, *therapeutic alliance*, *relationship* refer to a relational dimension. In particular, can observe the recurrence of verbs that express different modalities and approaches with respect to the interactions and motivations that can connote an interpersonal relationship. The terms *time*, *to happen*, *hourly*, and *work* evoke the temporal dimension of the therapeutic relationship.
- Cluster 4, named *Awareness*, aggregates 262 elemental contexts of the 1682 classified, which correspond to 15.58% of the variance (see Table 2).
- For this cluster the recurrence of the words *to understand*, *sense*, *path*, evokes a procedural aspect of understanding what happened; the same adverb *probably* refers to a retrospective reasoning with respect to hypotheses and reflections. Even the terms such as *bound*, and *to tie* can refer to phrases that refer to the connection between events, precisely *linked* to each other and that can be put in relation.
- Cluster 5, named *Dropout*, aggregates 276 elemental contexts of the 1682 classified, which correspond to 16.41% of the variance (see Table 2).
- In this cluster the presence of terms such as *impasse*, *to end*, *before*, *last*, *session* specifically evokes the psychotherapeutic treatment and the relative stalemate with the consequent early interruption.

### Distribution Analysis

We proceeded with the analysis of the distribution of the five clusters among the different levels of the following variables: Diagnosis, Orientation, Supervision, and Triangulation.

As for the Diagnosis variable, the Axis I diagnosis is explained for its 25.8% variance from cluster 1, 18.7% from cluster 2, 23.5% from cluster 3, 14.1% from cluster 4 and 17.8% from cluster 5. With reference to the diagnosis on Axis II the variance is explained at 28.2% from cluster 1, 17.3% from cluster 2, 22.2 from cluster 3, 18.4% from cluster 4 and 14% from cluster 5.

Compared to the Orientation variable, the cognitive-behavioral orientation sees its variance explained to 20.7% by cluster 1, to 19.2% by cluster 2, to 22.1% by cluster 3, to 20.7% by cluster 4 and to 17.3% by cluster 5; for the psychodynamic orientation cluster 1 explains to 29.2% of the variance, 15.7% the cluster 2, the 22.3% from the cluster 3, 14.7% the cluster 4 and 18.1% from the cluster 5. The variance for the other psychotherapeutic orientations is the following: 30.1% the cluster 1, 19.3% the cluster 2%, 26.4% the cluster 3, the 10.8% the cluster 4 and the 13.5% the cluster 5.

Compared to the Supervision variable, for cluster of therapists who performed it, cluster 1 explains 36.3% of variance, 15.2% cluster 2, 22.1% from cluster 3, 12.6% cluster 4 and 13.9% from cluster 5. The variance for the group that did not perform post dropout supervision is explained with these results: 19.1% cluster 1, 20.2% cluster 2, 24.6% cluster 3, 17.9% cluster 4 and 18.3% cluster 5.

Compared to the Triangulation variable, for the group of cases in which the presence of this variable did not occur, cluster 1 explains to 25.8% of the variance, 17.5% the cluster 2, 24.8% from the cluster 3, 15% the cluster 4 and 17% from cluster 5. The variance for the group in which there was a triangulation is explained with these results: 29% cluster 1, 19.8% cluster 2, 19.1% cluster 3, 17.6% cluster 4, and 14.5% cluster 5.

### Correspondence Analysis

The correspondence analysis, aimed at comparing different segments of the corpus, was performed for the Orientation, Diagnosis and Communication variables (test threshold value for significance ±1.96).

#### Orientation

**FIGURE 2:**
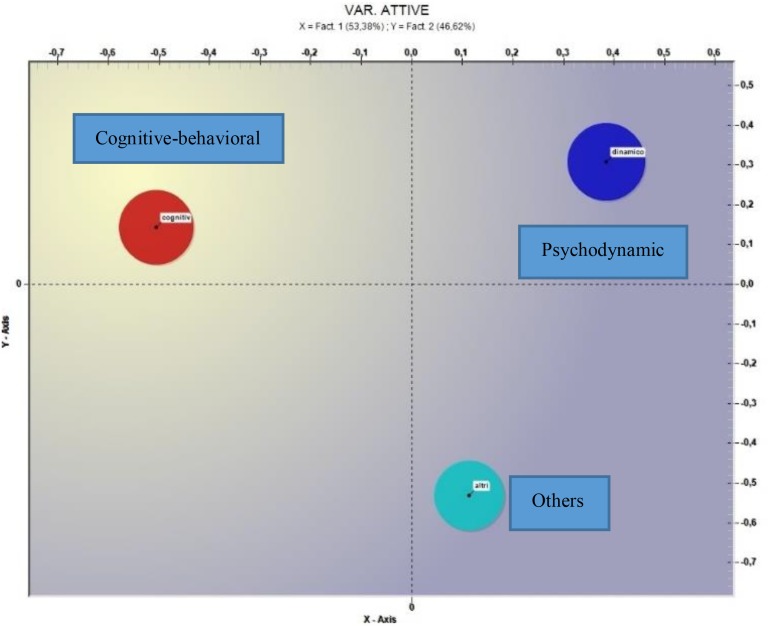
Correspondence analysis (*Variable = Orientation*).

The correspondence analysis for the Orientation variable (see Figure 2) showed two factors explaining, respectively, 53.38% and 46.62% of the data variance.

With respect to the first factor (i.e., horizontal axis) the negative factorial polarity shows a test value for the Cognitive-behavioral orientation of −46.12, for the positive factorial polarity the value of the test for the Psychodynamic orientation is 36.57 and for the Other orientations of 9.40; in Table 3 the most significant terms and the respective test values can be found.

**Table 3:** Correspondence analysis – therapeutic orientation.

Horizontal axis				Vertical axis			

Negative polarity		Positive polarity		Negative polarity		Positive polarity	
Lemma	Test value	Lemma	Test value	Lemma	Test value	Lemma	Test value
Cogn-behavior	−46.12	Psychodynamic	36.57	Others	−44.16	Psychodynamic	29.32
Sensation	−4.13	Others	9.40	Speech	−5.15	Cogn-behavior	13.03
Uncle	−4.15	Stuff	4.36	She	−5.53	Parent	4.16
Own	−4.27	Sons	4.06	Problem	−5.66	Comes	3.63
You	−4.73	Skip	3.57	To lose	−4.59	Sons	3.48
Probably	−4.85	Become	3.55	Receive	−4.68	Search for	3.11
Father in law	−4.98	Happen	3.43	Return	−4.74	Supervisor	3.04
Exit	−3.19	Partner	3.37	Work	−4.37	To start	3.00
To determine	−3.26	Separation	3.36	Ache	−3.27	Separation	2.88
Emotion	−3.26	Times	3.21	For me	−3.12	Little boy	2.88
Phobic	−3.26	Shoe	3.21	Method	−3.14	Work	2.87

The correspondence analysis with respect to the vertical axis shows a test value for the others mode of −44.16 for the negative factorial polarity; the test value for the Psychodynamic orientation is 29.32 and the Cognitive-behavioral of 13.03 for the positive factorial polarity. In Table 3 the most significant terms and the respective test values.

#### Diagnosis

**FIGURE 3:**
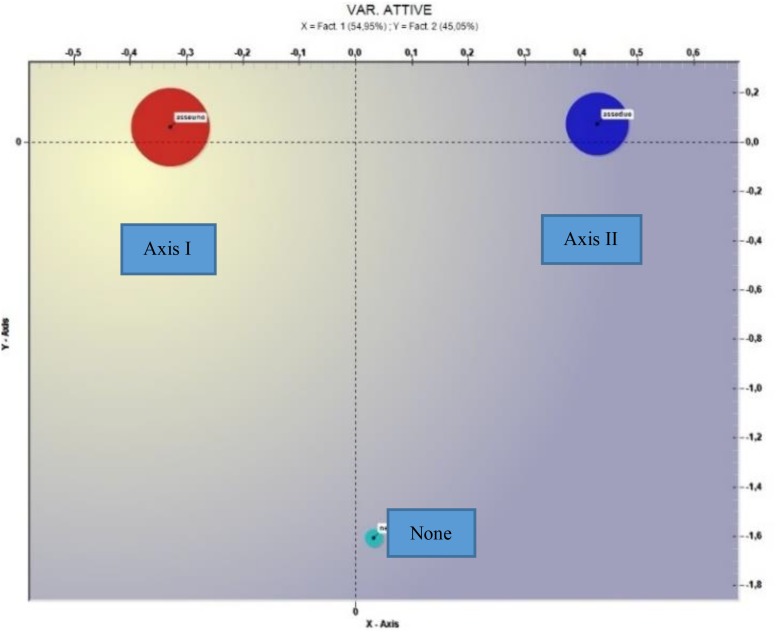
Correspondence analysis (*Variable = Diagnosis*).

The correspondence analysis for the Diagnosis variable (see Figure 3) showed two factors explaining, respectively, 54.95 and 45.05% of the data variance.

With respect to the horizontal axis the negative factorial polarity shows a text value of Axis I – 45.53; the value of the test of Axis II is 45.70 for the positive farm polarity. With respect to the vertical axis it produces a test value for the mode No diagnosis of −42.15 for the negative polarity; the test value for Axis I is 8.80 and for Axis II it is 8.07 for the positive polarity. In Table 4 the most significant terms and the respective test values.

**Table 4:** Correspondence analysis – diagnosis.

Horizontal axis				Vertical axis			

Negative polarity		Positive polarity		Negative polarity		Positive polarity	
Lemma	Test value	Lemma	Test value	Lemma	Test value	Lemma	Test value
Axis I	−45.53	Axis II	45.70	No diagnosis	−42.15	Axis I	8.80
Parent	−5.89	Own	9.40	Wife	−10.34	Axis II	8.07
Child	−5.04	Father in law	5.70	Emotion	−9.79	To success	2.43
Availability	−5.08	Obviously	4.36	To ask	−8.58	Years	2.12
To happen	−3.30	Return	3.78	You	−8.60	To understand	2.06
Appointment	−3.37	Boy	3.69	Way of doing	−7.15		
Know	−3.45	Stay	3.62	Shortly before	−7.15		
Play	−3.54	Not no	3.55	I remember	−7.65		
Clearly	−3.54	Borderline	3.50	Today	−6.41		
To find	−3.63	Front	3.47	To save	−6.54		
Life	−3.83	To leave	3.26	Ok	−5.08		

#### Communication

**FIGURE 4:**
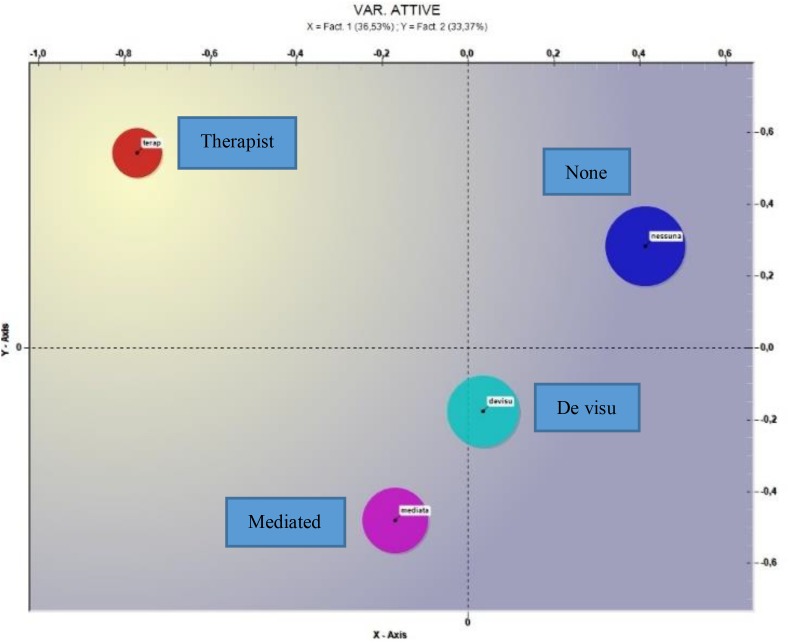
Correspondence analysis (*Variable = Communication*).

The correspondence analysis for the communication variable showed three factors; we considered the first two, explaining, respectively, 36.53 and 33.37% of the data variance (see Figure 4).

With respect to the horizontal axis the negative factorial polarity shows a test value for the therapist mode of −38.84 and −12.27 for the Mediated mode, for the positive factorial polarity it produces for the No communication mode a test value of 36.91. With respect to the vertical axis, the negative factorial polarity produces a test value of −34.79 for the Mediated mode and for the *de visu* mode of −14.05, for the positive factorial polarity it produces a test value for the Therapist mode of 27.38 and for the modality No communication of 25.32. In Table 5 the most significant terms and the respective test values can be found.

**Table 5:** Correspondence analysis – communication.

Horizontal axis				Vertical axis			

Negative polarity		Positive polarity		Negative polarity		Positive polarity	
Lemma	Test value	Lemma	Test value	Lemma	Test value	Lemma	Test value
Therapist	−38.84	No communication	36.91	Mediated	−34.79	Therapist	27.38
Mediated	−12.27	Respect	5.59	*De visu*	−14.05	No communication	25.32
Error	−5.01	Stuff	4.30	Receive	−4.06	Fifteen	5.24
Borderline	−5.23	Appointment	4.27	Finance	−4.33	Uncle	4.79
Problem	−5.33	In mind	4.16	Pharmacological support	−3.05	You	4.39
Admitted	−4.05	Father in law	4.05	Confusion	−3.11	To escape	3.68
Money	−4.08	I remember	3.79	Record	−3.13	Community	3.66
Thirteen	−4.09	Dream	3.78	Employee	−3.17	Phobic	3.66
Graduate	−4.16	Sensation	3.76	Important	−3.20	Failure	3.49
To escape	−4.18	Little boy	3.75	Return	−3.26	Appointment	3.44
Be sorry	−4.23	Comes	3.72	Easy	−3.37	Similar	3.34
She	−4.28			of _this_type	−3.40	Punch	3.34

### Specificity Analysis

Finally, the specificity analysis on the Supervision and Triangulation variables was carried out. Below are the results that refer to the therapists who have carried out post-dropout supervision and to those who have not done so. Table 6 shows the exclusive lexical units of Supervision or No supervision group comparing the whole corpus and their occurrence.

**Table 6:** Exclusive specificity analysis – no supervision group vs. supervision group.

No supervision		Supervision	
Lemma	Occurrence	Lemma	Occurrence
Father in law	14	Serious	13
Uncle	13	Return	10
Thing	11	Structure	9
To avoid	9	Dog	7
Own	9	Machine	7
Request	9	Ugly	6
April	8	Community	6
Mate	8	dark_Queen	6
Component	8	To exist	6
Guide	8	Pregnancy	6
Improvement	8	Abandonment	5
Avoidance	7	Hospitality	5
In discussion	7	Activated	5
Schedule	7	Included	5
To escape	7	Depressed	5
Alternative	6	Distraction	5
Borders	6	Paranoid_disorder	5
Emotion	6	Letter	5
Phobic	6	Improve	5
Frankly	6	Permit	5

Table 7 shows the results, relative to the presence/absence mode of post dropout Supervision, compared to the typical lexical units; chi-square (threshold value χ^2^ = 3.84, *df* = 1, *p* = 0.05).

**Table 7:** Typical specificity analysis – no supervision group vs. supervision group.

No supervision		Supervision	
Lemma	χ^2^	Lemma	χ^2^
She	37.91	To ask	16.07
Mom	22.65	Comes	13.24
I think	22.19	Speech	13.24
Supervision	18.18	Path	12.32
Anger	17.99	Stuff	11.36
Receive	16.47	Clearly	10.19
Own	16.40	In mind	9.89
Jump	15.73	To find	9.55
Sitting	13.38	Sensation	8.78
Psychiatrist	11.82	Latest	8.75
Patient	11.81	Appointment	7.74
Little boy	10.62	Positive	7.60
Drug	9.44	Wife	7.56
Obviously	9.43	Idea	7.49
Live	8.72	Different	7.36
Difficult	8.40	Real	7.28
Pharmacological support	8.30	Era	7.28
Confusion	8.23	Place	7.28
Scare	8.23	Previous one	7.28
Report	7.79	Today	7.04

The results of the analysis of the specificities comparing to the cases in which a triangulation was present between the variables associated with the impasse can be found below. In Table 8 the exclusive lexical units and their occurrence are presented.

**Table 8:** Exclusive specificity analysis – triangulation group vs. no triangulation group.

Triangulation		No triangulation	
Lemma	Occurrence	Lemma	Occurrence
Father in law	14	Error	24
Little boy	12	Brother	20
Shoe	10	To pay	20
Eighteen	7	To introduce	17
Borders	6	Place	13
Reconnect	6	Expectation	12
Constitute	5	Panic attacks	12
Distraction	5	Staff	12
Cannabis	4	To disappear	12
Setting up	4	Approach	11
Maturity	4	Position	11
At the same time	4	Space	11
Neuroscience	4	Confusion	10
nutritionist	4	Trust	10
potere_AMB	4	Return	10
Shame	4	Sorry	10
		Excuse	10
		Money	10
		Anxious	9
		Elaborate on	9

Table 9 shows the results, relative to the presence/absence mode of Triangulation, with respect to the typical lexical units; chi square (threshold value χ^2^ = 3.84; *df* = 1; *p* = 0.05).

**Table 9:** Typical specificity analysis – triangulation group vs. no triangulation group.

Triangulation		No triangulation	
Lemma	χ^2^	Lemma	χ^2^
Parent	66.16	I	25.87
Speech	35.82	You	20.89
Child	22.83	For me	12.05
Sensation	22.07	We	8.81
Involve	20.73	To ask	8.74
Of this type	20.65	Time	8.69
Therapy	19.91	Ok	8.50
Mate	18.23	Stuff	8.22
Of course	16.85	Person	7.73
Indoor	15.45	Fear	6.83

## Discussion

There are four countertransference responses that are on average more stressed than the other 5; in order: Helpless/Inadequate, Parental/Protective, Positive/Satisfying, and Overwhelmed/Disorganized. The reaction and the most widespread feelings by the therapist in the dropout cases seem to be those of inadequacy, incompetence and anxiety; the awareness of being in a deadlock and the inability or impossibility to solve it could have led to this experience. The second most urged factor is the Parental/Protective: the therapist feels invested in a parental role and feels compelled to take care of the patient in ways that exceed what may be normal positive feelings toward the patient. This factor, moreover, as noted by Betan et al. (2005), is positively correlated with personality disorders of DSM-IV TR cluster C: avoidant, obsessive-compulsive, and dependent (Sanavio and Cornoldi, 2001). Therefore, it is hypothesized that the above-average presence of the group of this factor can be connected to the presence of incorrectly diagnosed cluster C personality disorders.

In this sense it is interesting to point out that the disorders of cluster C are the most widespread in the general population (Torgersen et al., 2001; Coid et al., 2006; Torgersen, 2014); with respect to the clinical population, the most widespread personality disorder is borderline (*M* = 28.5%), the second is avoidant (*M* = 24.6%), and the third is dependent (*M* = 15%) (Torgersen, 2014). It is emphasized for the present discussion that the disorders of the Cluster C are characterized by a strong difficulty in the management of work and in establishing and maintaining intimate affective relationships. This seems congruent with the general background of the patients examined that are characterized by a past of difficulties in interpersonal relationships. Furthermore, the presence of a personality disorder in this cluster is associated with worse outcomes in the treatment of Axis I disorders (Shea et al., 1990; Reich and Vasile, 1993). This could partly explain the dropouts even in cases where the patient has shown a psychopathological picture in the first instance afferent to Axis I disorders.

Comparing to the cases examined, the therapist seems to try, even more than the average of the other factors, feelings of satisfaction (Positive/Satisfying factor) and a conviction with respect to the good course the therapy; the patient himself could contribute to this countertransference response, with the formal adhesion to the therapeutic path and with a compliant attitude and acquiescent behaviors toward the therapist. It could indeed be only a phase of treatment: the reference is to that positive period of progress that often precedes a stalemate, but which is actually fueled by the patient’s transference toward the therapist and not by a real change and transformation of the patient (Freud, 1915–1917).

This would be an impasse of “withdrawal,” that according to Safran et al. (2014) occurs when the patient shows excessive compliance or fails to express his difficulties. Another aspect to be taken into account is that the Positive/Satisfying countertransference activation could be linked to the often incorrect assumptions with respect to the degree of satisfaction (Norcross, 2005). Therefore, the feeling that there is a good therapeutic alliance could actually conceal a misalignment with respect to objectives and strategies. Another explanation of the activation of this factor above the group average could be linked to the positive re-evaluation of the impasse event, possibly influenced also from the time passed between the completion of the questionnaire and the dropout case described.

Finally, the fourth emotional response of the most stressed therapist refers to the Disorganized/Overwhelmed factor and shows how the therapist experiences strong feelings of repulsion and resentment toward the patient and wishes to escape from it. On one hand, this could be influenced by the stressful situation to which the therapist is subjected and on the other could be linked to one of the patterns that the patient unconsciously evokes in the therapist (Betan et al., 2005), prompted in this case to confirm possible expectations of rejection by the patient (Ogden, 1982; Gabbard, 2001).

The analyses on the presence of differences among therapist’s countertransference responses according to some structural variables confirm only partially the hypotheses. Indeed, there were not significant differences related to the therapist (gender and orientation) variables as well as to the patient’s diagnosis. On the contrary, a significant difference emerged for the patient’s gender with female patients eliciting less sexual feelings in the therapist than the group of male patients. However, this finding could be explained by the gender imbalance in the sample of therapists consisting of 21 women and only 7 men and it requires further investigation.

Although only a significant difference between male and female patients emerged for sexualized countertransference, overall the relevance of transference in dropout situations is underlined by the present research, as already demonstrated by other studies (Weiner, 1974; Atwood et al., 1989; Elkind, 1992; Nathanson, 1992; Pulver, 1992; Hill et al., 1996). Indeed, therapists in stalemate situations report having difficulty in managing and containing what are the patient’s negative affects, as can be closely linked to the failure to overcome past situations or to opposing and provocative behavior even with a difficult management of the setting. This last aspect is one of the characteristics of the Special/Overinvolved countertransference, which presents in the whole group of therapists values below the general average. Some themes related to countertransference seem to play a role; the therapists recognize elements that can be traced back to their personal history and to past experiences reactivated by treatment with the patient; there is an excessive desire to achieve good intervention and good clinical performance. This therapist’s personal expectation may have affected the moment they put the centrality of the patient and the therapy in the background. This aspect would seem to be linked to what is detected and defined in the research by Hill et al. (1996) as a fixation on the so-called role of the savior, in which the therapist feels he must “save” his patient and take care of it at all costs. These stresses are attributed to personal motivations by a therapist who has influenced the choice of a helping profession.

On this theme Miller (1996) maintains that there is a common past for those who choose the profession of psychotherapist; in his opinion, those who practice this discipline have developed “a special sensitivity for the unconscious signals of the needs of others” because they have been the object of satisfying of the parents’ emotional needs during childhood. For Miller, in the past of those who practice the profession is “always present a deeply insecure mother on the emotional level, which for her own emotional balance depended on a certain behavior or way of being of the child” (ibid., P.15). With time, the child to ensure his parents’ love refines his ability to respond and be supportive (Miller, 1996).

From the analysis carried out with T-LAB, the five thematic clusters that emerged offer interesting food for thought. As it also emerges on a graphical level, the thematic nuclei produced could be grouped into three large thematic areas, given the spatial contiguity between them.

(1) Cluster 1 – *Mother figure* and cluster 3 – *Relationship configuration* could constitute a *first area* that probably evokes a transference or countertransference theme linked in a salient way to a female figure.
(2) A *second thematic area* is the one related to cluster 2 – *Diagnosis* linked specifically to the pathology and its severity. This aspect plays an important role in the light of clinical studies in which it has been seen that patients with severe symptoms tend to activate negative emotional responses in the psychotherapist, as well as a considerable difficulty in the construction of the therapeutic alliance (Bender, 2005; Røssberg et al., 2010; Dahl et al., 2012, 2014; Lingiardi and McWilliams, 2015; Tanzilli et al., 2016).
(3) The *third area*, which sees the cluster contiguous – 4 *Awareness* and the cluster 5 – *Dropout*, could refer to the procedural aspects and the involution of the impasse situation; in this sense, therapists could highlight the difficulty in managing the stalemate or in the ability to identify precursory signals or events that can signal the impasse in time. In this sense, as underlined by Safran et al. (2011), the training of therapists in the ability to identify the impasse and to treat them in a constructive way has a positive impact on the outcomes of the therapy.

The analysis of the correspondences with respect to orientation shows with the bi-polarity on the *X*-axis that therapists with a cognitive-behavioral orientation use a language that is significantly different from psychodynamic therapists and other orientations. The terms *sensation*, *emotion* and *exit* used by the first evoke, compared to situations of impasse, a more instinctive or *belly* approach than the approach that can be inferred from the terms used by therapists with other guidelines. The latter, as a matter of fact, seem to have manifested a minor urgency and perhaps it seems to have a privilege, before a separation from the impasse, a request for support to face the stalled phase of the therapy (*search for*, *supervisor*, *to start*, *work*).

Compared to the *Y*-axis, therapists of other orientations seem to express greater emotional and relational involvement (*she*, *problem*, *to lose*, *receive*, *pain*) compared to cognitive-behavioral therapists and psychodynamic one, who seem to be more focused on the family context (*parent*, *children*, *little boy*). They could also represent different strategies to overcome the impasse; in the first case with the focus on the specific dynamics of the dyad and in the second one with an analysis of the influences on the treatment of the patient’s family context.

As far as the diagnosis variable is concerned, from the bi-polarity on the *X*-axis it emerges that the language, compared to an axis I diagnosis, is significantly different from that in the presence of a patient diagnosed on axis II. In particular, it seems that in the presence of a diagnosis from axis I there is a more positive climate, as words like *availability*, *appointment*, *play*, *know*, *life* shown; in the second case the terms *to leave*, *front*, *borderline* seem to indicate a more negatively connoted climate.

With respect to the different modes of communication of the interruption, where this has occurred in a mediated or communicated way by the therapist, an atmosphere of urgency or gravity from the frequency of the lemmas (*error*, *Borderline*, *admitted*, *to escape*, *be sorry*) emerges. In the case in which there has not been the patient’s communication, the theme of respect, which would have failed with this gesture of the patient toward the therapist and also the theme of the search for meaning of the patient’s behavior, is emerged (*in_mind*, *remember*, *dream*).

There is also a significant difference between the Mediated and *de visu* modes on one side and the Therapist and No Communication on the other. In the first case it evokes a more positive and comprehensive context or situation (*receive*, *important*, *easy*, *return*), while in the second one it seems that the two modalities express a critical and urgent situation (*to escape*, *failure*, *punch*).

Finally, the verification of the relationships between the five clusters and the variables does not seem to detect significant data regarding the Diagnosis, Orientation and Triangulation variables. On the other hand, it seems interesting to point out that compared to the therapists who supervised the impasse, cluster 1 – *Mother Configurations* explains almost twice the variance compared to the group of those who did not use it. It seems then that the salience and intensity of this theme pushes the therapist to request help or advice post dropout. This behavior could suggest that the evoked female figure is therefore more referable to the therapist and therefore to more aspects of the countertransference. In favor of this hypothesis also the comparison of the typical lexical unit among the therapists who have used supervision, for which we highlight the most significant values for the words *she*, *mother*, *I think*, *supervision* and those who have not carried it out for which the most salient terms evoke more perhaps an intent than an urgency (*to ask*, *speech*, *path*) gives us some evidence.

Lastly, also for the variable Triangulation we highlight, in the comparison of the typical lexical unit with dropouts in which no intrusion was signaled by a third party, the presence of the words *parent*, *child*, *speech*, *involve* that can signal on one hand the importance of parental figures as variables associated with the impasse and on other hand also the need for a more participated and careful management of this third “external” to the therapeutic dyad. It is interesting to observe that the absence of triangulation is well evidenced by the typical occurrence of lexical units such as *I*, *you*, *us*; therefore, it seems that the intrusion of a person external to therapy may have a specific weight and importance in determining the impasse situation and the consequent drop out, as highlighted above also by the percentages found in the sample.

### Limits and Future Developments

The *first limit* of this study is related to the number of the sample, which does not allow generalizations with respect to which are the most stressed countertransference in cases of dropout and the lack of a control group. The results of the present research should therefore be repeated on a sample with a higher number of subjects and should be compared with cases terminated with a positive result.

A *second limit* is linked to the possible memory bias of the therapists on the cases examined; as a matter of fact, the clinicians were invited to fill in the questionnaire on their emotional responses to remember how they had felt most of the time with the patient described in the interview. Lacking a homogeneity for the therapists with respect to the time between the compilation of the TRQ and the end of the treatment of the patient examined, it can be hypothesized that this has influenced both the quality of the evocation of the emotional aspects of the experience and the details of the same.

A *third limit* also reported in the research by Hill et al. (1996) is linked to the partial perspective with respect to the impasse examined. The point of view and the experience analyzed is only that of the therapist. What we know of the patient’s experience is reported by the clinician and not by him in the first person. In this sense, it could be interesting to compare the experience of both components of the therapeutic dyad with respect to a dropout (Hill et al., 1996).

Finally, for the present work a further implementation of the analysis of the corpus composed by the interviews seems interesting in terms of development. Through a more in-depth refinement of the text preparation procedures (such as greater disambiguation), we could found out other interesting elements that characterize the impasse situations and other variables associated with the deadlock situations.

In this sense, identifying other possible factors or evaluating the weight that everyone can have in terms of effects and how they interact in contributing to premature interruptions of psychotherapy seem important for future research. Finally, a greater diffusion of the results of the researches dealing with this topic is also hoped for; certainly useful in the prevention and identification of those events that precede the phenomenon of dropout, as pointed out by several authors.

## Ethics Statement

This study was carried out in accordance with the recommendations of the Ethical Committee of the Catholic University of Sacred Heart of Milan. All subjects gave written informed consent in accordance with the Declaration of Helsinki.

## Author Contributions

SiM conceived and designed the study, collected and interpreted the data, and wrote the manuscript. SaM analyzed and interpreted the data, and reviewed the manuscript. OO developed the study design, contributed to the interpretation of the data, and wrote and reviewed the manuscript.

## Conflict of Interest Statement

The authors declare that the research was conducted in the absence of any commercial or financial relationships that could be construed as a potential conflict of interest.

## Footnotes

1 transcribe.wreally.com
